# Development of Efficient Genome-Reduction Tool Based on Cre/*loxP* System in *Rhodococcus erythropolis*

**DOI:** 10.3390/microorganisms11020268

**Published:** 2023-01-19

**Authors:** Wataru Kitagawa, Miyako Hata

**Affiliations:** 1 Bioproduction Research Institute, National Institute of Advanced Industrial and Technology (AIST), Sapporo 062-8517, Japan; 2 Graduate School of Agriculture, Hokkaido University, Sapporo 060-8589, Japan

**Keywords:** *Rhodococcus*, genome reduction, Cre/*loxP*, genetic tool, plasmid curing

## Abstract

*Rhodococcus* has been extensively studied for its excellent ability to degrade artificial chemicals and its capability to synthesize biosurfactants and antibiotics. In recent years, studies have attempted to use *Rhodococcus* as a gene expression host. Various genetic tools, such as plasmid vectors, transposon mutagenesis, and gene disruption methods have been developed for use in *Rhodococcus*; however, no effective method has been reported for performing large-size genome reduction. Therefore, the present study developed an effective plasmid-curing method using the levansucrase-encoding *sacB* gene and a simple two-step genome-reduction method using a modified Cre/*loxP* system. For the results, *R. erythropolis* JCM 2895 was used as the model; a mutant strain that cured all four plasmids and deleted seven chromosomal regions was successfully obtained in this study. The total DNA deletion size was >600 kb, which corresponds mostly to 10% of the genome size. Using this method, a genome-structure-stabilized and unfavorable gene/function-lacking host strain can be created in *Rhodococcus*. This genetic tool will help develop and improve *Rhodococcus* strains for various industrial and environmental applications.

## 1. Introduction

The genus *Rhodococcus* is a Gram-positive bacterium with a high G+C content belonging to the phylum Actinobacteria and has been evaluated in various aspects, such as ecology, pathogenicity, biosurfactant production, and degradation of chemical compounds [1,2,3,4,5,6]. In particular, the ability to degrade chemicals, especially aromatic compounds, has been extensively studied, and this is the most distinguishing feature of *Rhodococcus* [7,8,9]. In recent years, antibiotic-producing strains and novel bioactive compounds have been obtained from this genus [10,11,12].

At present, *Rhodococcus* has been isolated from a wide range of environments, including freshwater, seawater, soil, deep-sea floors, and polar regions as well as from animals, plants, and insects [5,13,14,15,16,17]. The diverse activities of this genus are associated with the wide range of environments colonized. The diverse activities of *Rhodococcus* are attributed to the genes possessed. Recently, many draft and complete genomes have been analyzed, and the chromosome sizes have been reported to be 6–7 Mb; the presence of linear and/or circular plasmids results in an increase in genome size of 1–2 Mb [18,19,20]. The large-sized genome structure can accommodate non-essential genes, and it makes this genus highly versatile.

A characteristic feature of the genomic structure of *Rhodococcus* involves its topology; certain species have circular chromosomes, and others have linear chromosomes. Four strains of this genus, including *R. jostii* RHA1 [20], *R. opacus* B4 [21], *Rhodococcus* sp. Strain R79, and *R. koreensis* strain R85 [19], have been clearly shown to possess a linear chromosome on the basis of complete genome sequencing analysis. Most of the other genomes analyzed in strains including *R. opacus* PD630 [18], *R. fascians* D188 [22], *R. rhodochrous* EP4 [23], *R. equi* strain DSSKP-R-001 [24], *R. ruber* strain C1 [25], *R. triatomae* DSM 44,893 [26], *R. globerulus* strain D757 [27], and *R. erythropolis* JCM 2895 [28] have been shown to possess a circular chromosome. The presence/emergence of a linear chromosome in Actinobacteria has been attributed to recombination between the circular chromosome and linear plasmid [29,30]. In this theory, the whole chromosomal DNA is integrated into the linear plasmid, and it behaves as a single linear replicon in this irreversible transformation. Therefore, the difference in linear or circular chromosomes is not related to the evolutionary lineage, and it most probably occurs individually in each strain. This phenomenon may occur at any time in strains containing linear plasmids, between both circular and linear replicons.

The presence of plasmids in *Rhodococcus* is of great ecological and functional importance. In *R. equi* and *R. fascians*, genes related to pathogenicity and infection are located on plasmids [3,31,32]; in *R. jostii* RHA1, *R. erythropolis* TA421, and other strains, the aromatic compound degradation genes are located on a large linear plasmid in addition to the chromosome [33,34,35]. In *R. erythropolis* BD2, the genes associated with metal tolerance are located on a linear plasmid [36]. In addition, *R. erythropolis* JCM 6824 contains antibiotic biosynthesis genes, which are located on the chromosome [37,38]. In contrast, antibiotic biosynthesis genes are encoded in a small circular plasmid in *R. erythropolis* JCM 2895 [28,39]. Regardless of the topology and size, plasmids are important for various functions in this genus.

Based on the activities of *Rhodococcus*, it has been developed and used as a microbial host, especially for mediating the biodegradation and bioproduction of certain compounds. Several genetic tools have been developed at present [40,41,42], and highly effective codon optimization techniques have been demonstrated in the genus to increase the expression level of cloned genes [43]. The advantages of using *Rhodococcus* as a microbial host, in addition to its activities mentioned above, are listed as follows: it grows quickly among Actinomycetes species and shows optimal growth on conventional nutrient medium such as Luria-Bertani (LB) agar. It does not have a life cycle (differentiation, such as sporulation) which is generally observed in *Streptomyces*, and it can be handled in the same manner as *Escherichia coli*. Since *Rhodococcus* is GC-rich, it is naturally suitable for expressing genes of *Streptomyces* and other Actinomycetes, which possess several genes that encode secondary metabolites. Useful genetic tools/methods have been established, including multiple plasmid vectors [44], gene-disruption systems via homologous recombination [40], transposon mutagenesis [45], and effective electrotransformation [46].

Genomic instability, which is an undesirable trait for a host microorganism, has been observed in *Rhodococcus*. For example, the emergence of alternative-sized linear plasmids has been observed in *R. jostti* RHA1 and *R. opacus* DSM 43,250 during laboratory cultivation [47,48]. The aforementioned chromosomal linearization is also associated with genomic instability. In addition, size reduction of circular plasmid vectors introduced in genetic experiments has been observed in several species (laboratory knowledge). This genomic instability may affect research experiments. Recent genome sequencing analysis of *Rhocococcus* identified several linear/circular plasmids; however, these cryptic plasmids are not essential in *Rhodococcus* used as a host microorganism based on compatibility with the vector to be introduced and the possibility of intra-genome recombination, as described above. Few studies reported that deletion of the plasmid improves the growth of the strain [49,50,51] or improves the expression of the gene in the vector [52]. Therefore, deletion of these cryptic plasmids is strongly desired.

The development of sophisticated host–vector systems using this genus remains an important area of study. An additional concern of using *Rhodococcus* as a host includes its innate abilities, such as bioproduction and degradation. Undesired background activity is anticipated when using the host for these purposes. In order to cancel the background activity, genes/gene clusters responsible for the activity must be deleted. These sizes are sometimes very large; therefore, conventional gene-disruption methods based on homologous recombination are not applicable. Genome engineering methods for large-scale deletion of chromosomal DNA, which greatly affects the genome structure, have not been established in *Rhodococcus* so far, and an effective method is desired [53,54].

This study reports an effective method for curing a cryptic plasmid and an effective genome-reduction method in *R. erythropolis*. The former contributes to increased genomic and introduced-vector stability, while the latter enables the removal of undesirable host innate genes and activities.

## 2. Materials and Methods

### 2.1. Bacterial Strains, Plasmids, Primers, and Culture Conditions

Bacterial strains and plasmids used in this study are listed in Table S1 and primers in Table S2. For both *Rhodococcus* and *E. coli* cultivation, LB medium was used and kanamycin (200 µg/mL for *Rhodococcus*, 25 µg/mL for *E. coli*), chloramphenicol (34 µg/mL), apramycin (50 µg/mL), ampicillin (100 µg/mL), and thiostrepton (0.5 µg/mL) were added when necessary. W-minimal medium [55] supplemented with succinate (0.2%, *w*/*v*), sucrose (0.2%, *w*/*v*), casamino acids (0.2%, *w*/*v*), and thiamine (0.1%, *w*/*v*) was used for preparation of electrocompetent *Rhodococcus*.

### 2.2. Electrotransformation

Electrocompetent cells were prepared as follows: *Rhodococcus* cells grown in the liquid medium were collected via centrifugation (at 8000× *g*, 5 min, 4 °C), and the cell pellets were washed twice with sterilized 10% glycerol, after which the cells were finally resuspended in 10% glycerol. The cells were directly used for the next step or stored in −80 °C. The plasmids were introduced into the cell using the Gene Pulser Xcell Electroporation System (Bio-Rad, Hercules, CA, USA) (setting parameters: exponential protocol, voltage: 1.6 kV/cm, resistance: 500–800 Ω, time constant: 9–11 ms). Recovering culture was statically incubated in liquid LB medium containing 10 mM glucose for 4 h at 28 °C. Cells were then spread on LB agar containing an appropriate antibiotic to select the transformants.

### 2.3. Plasmid Curing Using sucB Gene

**Figure 1:**
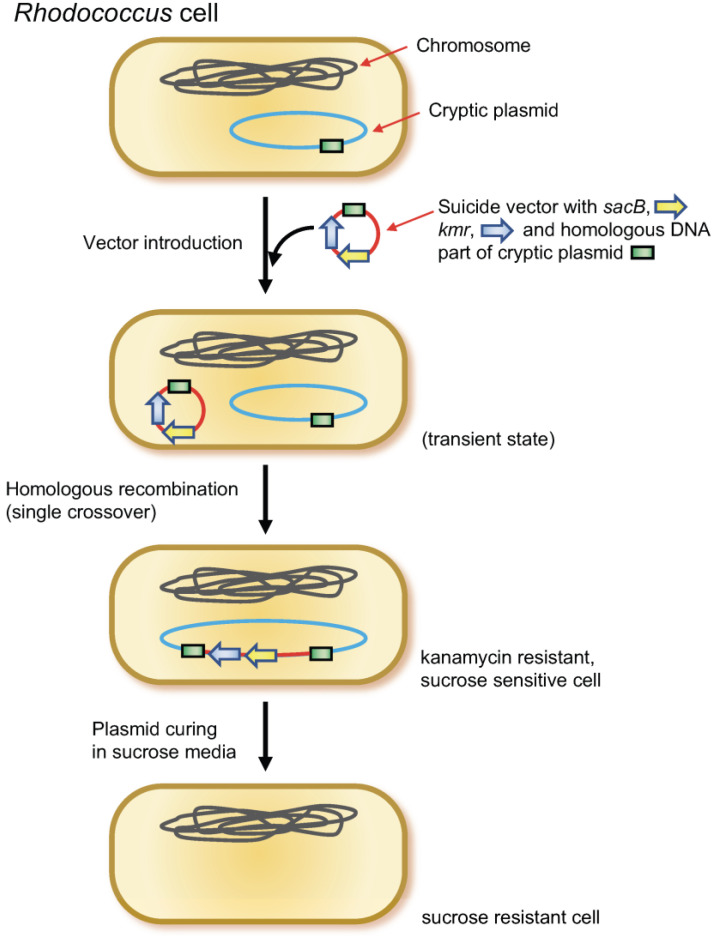
Plasmid-curing method using *sacB* gene. Suicide vector with *sacB* gene was integrated to the cryptic plasmid to be cured by homologous recombination. Subsequently, the plasmid was cured by cultivating in a media containing 20% sucrose.

The pK18mobsacB vector [40,56] (suicide vector in *Rhodococcus*) containing a homologous DNA region (approximately 3 kb, PCR-amplified) of curing target plasmid was introduced into *Rhodococcus* cells via electrotransformation. Subsequently, kanamycin-resistant colonies were selected on agar medium and confirmed for homologous recombination (single crossover) using PCR. Further single colony isolation on the kanamycin media was conducted when necessary. Subsequently, the mutant was cultivated in liquid LB without kanamycin, followed by spreading on LB agar containing 20% sucrose without kanamycin. The loss of target plasmid was confirmed via colony PCR using two or three independent primer sets, which amplified different parts of the target plasmid (Figure 1). The PrimeSTAR GXL DNA polymerase and Tks Gflex DNA Polymerase (Takara Bio, Ootu, Japan) were used for DNA cloning and colony PCR, respectively.

### 2.4. Genome Reduction Using Cre/loxP System

#### 2.4.1. Vector Construction

**Figure 2:**
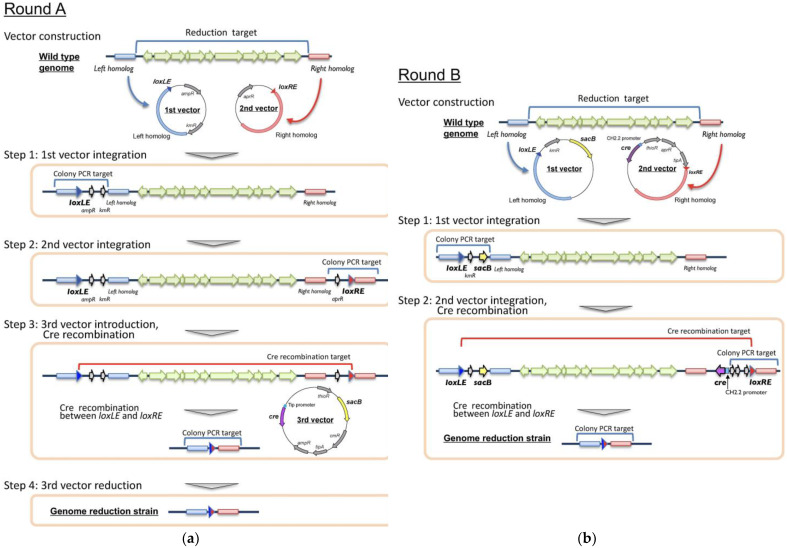
Large-size genome-reduction method using Cre/*loxP*. (**a**) Four-step procedure requires three plasmid-introduction and one plasmid-reduction steps (Round A). (**b**) Two-step procedure requires only two plasmid-introduction steps (Round B). See details in the text.

pBS-aphII-loxLE (used for the first vector construction in Round A) was constructed on the basis of pBluescriptII by introducing kanamycin-resistant gene (*aphII*) from pK18mobsacB and synthesized *loxLE* sequence (TACCGTTCGTATAGCATACATTATACGAAGTTAT) [57]. pACYC-aac-loxRE (used for the second vector construction in Round A) was constructed on the basis of pACYC184 [58] by introducing apramycin-resistant gene (*aac(3)IV*) from pHN1237 [59], synthesized *loxRE* sequence (ATAACTTCGTATAGCATACATTATACGAACGGTA) [57] and by deleting the tetracycline-resistant gene. pTip-sacB-cre (used as the third vector in Round A) was constructed on the basis of pTip-QC2 vector (*E. coli*-*Rhodococcus* shuttle vector [44]) by introducing *sacB* gene from pK18mobsacB and Cre recombinase gene (*cre*). The *cre* gene was placed under the regulation of thiostrepton-inducible Tip promoter [44]. pK18mobsacB-loxLE (used for the first vector construction in Round B) was constructed by introducing *loxL*E sequence into pK18mobsacB. pCH-cre-loxRE (used for the second vector construction in Round B) was constructed by ligating p15A ori from pACYC184, thiostrepton-inducible protein gene (*tipA*) and thiostrepton-resistant gene (*tsr*) from pTip-QC2, *aac(3)IV*, *cre*, and thiostrepton-inducible CH2.2 promoter from pTip-CH2.2 [41]. The *cre* gene was placed under the regulation of CH2.2 promoter. For each deletion target, the first vector was constructed by ligating an upstream flanking part of deletion target region (left homolog, approximately 3 kb), whereas the second vector was constructed by ligating a downstream flanking part of the deletion target region (right homolog, approximately 3 kb) (Figure 2).

#### 2.4.2. Genome-Reduction Strategy

Round A (Figure 2A): The first plasmid vector was introduced into the cells and a single-crossover mutant (kanamycin-resistant) was selected as described above. Subsequently, the second plasmid vector was introduced into the mutant and a second single-crossover mutant (kanamycin- and apramycin-resistant, single crossover at two positions) was selected. Next, the second-single crossover mutant was transformed using the third vector, and a chloramphenicol-resistant colony was selected. *cre* recombinase expression (on the third vector) was induced by adding thiostrepton (0.5 µg/mL) in liquid medium without other antibiotics, followed by spreading on LB agar. The deletion of the target region between *loxLE* and *loxRE* was confirmed via colony PCR. Finally, the third vector was cured by cultivating cells on LB agar containing 20% (*w*/*v*) sucrose without antibiotics. Round B (Figure 2B): The first and second vectors were introduced, and second-single crossover mutant was selected as in Round A. *cre* recombinase expression (on the second vector, genome-integrated) was induced by adding thiostrepton. The deletion mutant was selected by cultivating colonies on LB agar containing 20% sucrose, and the deletion was confirmed via colony PCR.

## 3. Results

### 3.1. Isolation of Plasmid-Cured Strains

*R. erythropolis* JCM 2895, one of the strains used in the present study, produces a bacteriocin-like antibiotic protein [39]. Since JCM 2895 shows faster growth and less biofilm/aggregate formation compared with that of other *R. erythropolis* strains, we selected it as the candidate host strain. The complete genome has recently been decoded, and one linear plasmid (pR09L01, 227,989 bp) and three circular plasmids (pR09C01, 79,600 bp; pREC01, 5420 bp; and pREC02, 5444 bp) have been identified in addition to the one chromosome [28]. We first attempted to cure the four plasmids from the strain. Several plasmid-curing methods have been established, including repeating high-temperature culture [60], protoplast regeneration [61], and culture with sodium dodecyl sulphate [62,63] or acridine orange [63,64]. Among these methods, repeated application of high-temperature culture and protoplast regeneration were unsuccessful in curing plasmids from this strain (data not shown).

Since *sacB* is effective as a marker for counter selection in *Rhodococcus* [40,65], plasmid curing using *sacB* was attempted in the strain, as illustrated in Figure 1 (see also Materials and Methods). First, to obtain a circular plasmid pR09C01-cured mutant, a DNA fragment of partial pR09C01 was PCR-amplified using a primer set of R09CPD-F1 and R09CPD-R1 (amplify 3078 bp), which was inserted into pK18mobsacB at the EcoRI-XbaI site, resulting in a vector pK18R09CPD1. This vector was introduced into the wild-type strain, which successfully yielded kanamycin-resistant mutants. The appropriate single crossover was confirmed via colony PCR using a primer set, R09CPD-SCC1 and aphII-UR. The former hybridizes to the upstream flanking region of the homologous sequence, and the latter hybridizes to the kanamycin-resistant gene, which amplifies 3737 bp. The single-crossover mutant was then liquid-cultured without kanamycin and spread on LB agar containing sucrose without kanamycin. The colonies on the media were transferred to fresh media after which the kanamycin sensitivity and sucrose resistance were verified again. Plasmid deletion was confirmed via colony PCR using the R09CPD-F1 and R09CPD-R1 primer sets and was also confirmed using another primer set that amplifies another region of pR09C01 (R09CPD-F2 and R09CPD-R2, which amplify the 3006 bp). In this study, most of the candidates grown on sucrose-containing media were successfully cured of pR09C01, and the resultant strain was named strain R0901.

Using strain R0901, curing of a linear plasmid pR09L01 was attempted in the same manner. The suicide vector containing a part of pR09L01, called pK18R09LPD01, was constructed and introduced into R0901. A pR09L01-cured mutant was obtained, confirmed, and named strain R0902. A pREC01-cured strain R0903 and pREC02-cured strain R0904 were also obtained in the same manner.

### 3.2. Development of Large Fragment Deletion (Genome-Reduction) Strategy Using the Cre/lox System

Various techniques have been developed for deleting single genes or short DNA regions (approximately > 10 kb) and have been widely used in *Rhodococcus*. The marker-less gene deletion system using *sacB* gene represents a major strategy for use in this genus [40,65]. However, no effective large-scale DNA deletion or introduction methods that enable genome-scale engineering has been successfully applied in *Rhodococcus*. The Cre/*loxP-*mediated genome-reduction method has already been successfully used in the Actinobacteria *Streptomyces* and *Corynebacterium* [57,66]. In the Cre/*loxP* system, Cre recombinase specifically recognizes the 34 nt *lox* sequence and catalyzes recombination. The basic reaction involves looping out (deleting) a DNA region between two distant *lox* sequences, leaving a single *lox* sequence [67]. It is a useful marker-less method that can be repeated many times (at many sites) at a high success rate. However, the procedure of the Cre/*loxP* system is laborious and time consuming; it involves a four-step procedure including three plasmid-introduction and one plasmid-reduction steps.

This four-step procedure, with a minor modification, was first attempted in *R. erythropolis* JCM 6824 [38,65], an aurachin RE antibiotic producer, to remove a 95 kb chromosomal DNA region containing its biosynthetic gene cluster (Round A, Figure 2A). The target region of 95 kb is not found in other strains of *R. erythropolis*; therefore, it has been regarded as a non-essential region and selected for deletion. The upstream flanking region of the deletion target (left homolog) was PCR-amplified using a primer set of 95k-LE-F2 and 95k-LE-R. The 3.1 kb amplicon was inserted into the XbaI-SphI site of pBS-aphII-loxLE, which yielded the first vector pBS-D95k-LE. Similarly, the downstream flanking region of the deletion target (right homolog) was PCR-amplified using a primer set of 95k-RE-F and 95k-RE-R2. The 3.2 kb amplicon was inserted into the SpeI-Sse8387I site of pACYC-aac-loxRE, which yielded the second vector pAC-D95k-RE. The first vector was introduced into wild-type JCM 6824 cells, and a kanamycin-resistant single-crossover mutant was obtained and confirmed via colony PCR, as performed in the plasmid-curing method (primer set; D95k-LE-CF and LE-DR, which amplify 3256 bp). The second vector was introduced into the single-crossover mutant cells of the first vector, and the apramycin-resistant mutant was obtained and crossover was confirmed via colony PCR (primer set; RE-UF and D95k-RE-CR, which amplify 3458 bp). Subsequently, the double mutant was transformed using the third vector, pTip-sacB-cre, which contained the *cre* recombinase and *sacB* genes. The resultant kanamycin/apramycin/chloramphenicol-resistant mutant was cultivated with thiostrepton to induce Cre recombinase expression. The recombination (genome reduction) between *loxLE* and *loxRE* was confirmed via colony PCR using a primer set comprising D95k-LE-CF and D95k-RE-CR. Using this primer set, a 6486 bp amplicon was obtained from the genome-reduction mutant, whereas no amplicon was obtained from the wild-type because the target size of >95 kb was too large to amplify via PCR. The third vector containing *sacB* was cured on sucrose-containing medium, as performed in the plasmid-curing method. Consequently, a genome-reduction strain was obtained. This is the first study to report a large-size genome-reduction method using the Cre/*loxP* system in *Rhodococcus.* The last vector deletion step was simplified owing to the use of the *sacB* system compared with that in the *Streptomyces*/*Corynebacterium* system; however, it is still a laborious method and must be further simplified.

Next, the two-step genome-reduction strategy needing only two vector-introduction steps without a plasmid-reduction step, was developed in *R. erythropolis* R0904, a plasmid-less derivative of *R. erythropolis* JCM 2895 constructed as described above (Round B, Figure 2B). Deletion target positions not conserved among other *R. erythropolis* strains were examined using GenomeMatcher software [68] (Figure S1). A total of seven regions, each having a size of >5 kb, were selected for deletion (Targets 1–7 = T1–T7). The left and right homolog sequences of T1 were PCR-amplified and introduced into the pK18mobsacB-loxLE and pCH-cre-loxRE vectors, respectively. The resultant vectors pSLE-R09T1 (first vector) and pCRE-R09T1 (second vector) were introduced into the R0904 cells, and single-crossover mutants were isolated as performed in Round A. The differences between Round A and Round B were as follows: *sacB* and *cre* recombinase genes in the third vector in Round A were separately integrated into the first and second vectors, respectively, in Round B. Further, *sacB* counter selection is used, not for plasmid reduction but for genome reduction. The kanamycin- and apramycin-resistant double mutant was then liquid cultured in the presence of thiostrepton, followed by spreading on agar plates containing sucrose. Genome reduction was confirmed via colony PCR similar to that in the Round A. We found that 100% of colonies on the plates had deleted T1 (approximately 117 kb in size, Table 1), and this strain was named R0905. Since the T2 size is relatively small (6.4 kb, Table 1), deletion was performed using a known marker-less double-crossover method [65]. Briefly, left and right homologs were tandemly inserted in the pK18mobsacB vector, and the resulting pK18-R09T2 vector was introduced into strain R0905. The double-crossover mutant was then isolated (independent of the Cre/*loxP* system) and named strain R0906. Using strain R0906, a T3 deletion mutant was isolated via a two-step procedure (Round B strategy) as in the T1 and named R0907. Similarly, all the other T4–T7 deletion mutants were isolated and named R0908–R0911, respectively (Table 1).

## 4. Discussion

The plasmid-curing method using *sacB* gene was first demonstrated in the genus *Rhodococcus*. Using *sacB* gene for curing cryptic plasmids have been tested in other bacteria [69,70]; however, they introduced the *sacB* gene by random transposon mutagenesis or using incompatible vectors to kick out the target cryptic plasmid. The former strategy required a screening step for the genuine mutant strain having transposon (*sacB* gene) on the target plasmid from the mutant library. The latter strategy required construction of a new vector incompatible with the target plasmid, that is, replication system of the target must be identified before the curing. After removing the target plasmid by incompatibility, the introduced vector must be cured via *sacB* selection system. In addition, multiple incompatible vectors have to be prepared when multiple plasmids are targeted. In our study, *sacB* gene was exclusively integrated into the target plasmid via homologous recombination using a suicide vector. Therefore, our procedure greatly reduced the laborious steps reported in the previous study. The successful isolation of strain R0904, which was cured of all the four plasmids, demonstrated that it is a highly effective method for use in *R. erythropolis*. In the process of curing pR09C01 and pR09L01 plasmid, a genuine single-crossover mutant strain was obtained without single colony isolation of the candidate colony. In contrast, for curing pREC01 and pREC02, the single colony isolation step was required on kanamycin-containing media. During the additional subculture, transient mutant cells harboring both original shape plasmid and single-crossover plasmid might be eliminated, and genuine single-crossover mutant cells were selected (not experimentally proven). The former two large plasmids, pR09C01 and pR09L01, are single copy or low copy, whereas the latter two small plasmids, pREC01 and pREC02, are multiple copy. The copy number difference might have affected the selection, i.e., the latter required the further selection. In this study, additional one-time single colony isolation was enough for the isolation of the mutant.

In the genome-reduction study, approx. 95 kb and 117 kb chromosomal deletions were carried out in *R. erythropolis* JCM 6825 and JCM 2895 (in the case of T1), respectively. These results demonstrated that the Cre/*loxP* system is useful for large-size genome reduction in *Rhodococcus*. In the strain JCM 2895, plasmid curing and genome reduction was performed repeatedly. The final deletion strain *R. erythropolis* R0911 was cured of all four linear and circular plasmids and showed seven deleted strain-specific DNA regions on the chromosome. The total DNA deletion size was >600 kb, which corresponds mostly to 10% of the genome size (Table 1). These results clearly demonstrated that the two-step procedure newly developed in this study represents a simple, effective, accurate, and repeatable tool for performing genome reduction. The new method greatly reduces the time and effort required because only two vector-introduction steps are needed, whereas three vector-introduction and one vector-reduction steps are required in the previous Cre/*loxP* method [57,66].

The key technology that enables this advanced technique involves the strictly regulated expression of *cre* recombinase present in the second vector. The CH2.2 promoter used in this study induces gene expression in the presence of thiostrepton; however, it strictly suppresses expression in the absence of the inducer with the TipA regulatory protein [41]. When a leaky promoter was used regardless of whether the level was low (such as Tip promoter recruited in the pTip-QC2 vector), the recombination between *loxLE* and *loxRE* occurred immediately after the introduction of the second vector and before the single-crossover event with the right homolog since Cre recombinase is highly active. In this case, the entire second vector DNA sequence would be integrated at the *loxLE* site and genome reduction would fail (Figure S2). Using 20% sucrose instead of 10% that is generally used is important for achieving *sacB* counter selection in this strain. Sucrose at 20% retards growth compared with that obtained at 10%; it eliminates false-positive clones efficiently, which may depend on the strain used.

The genome-reduction system greatly depends on single-crossover and Cre recombinase activity. Therefore, this system is applicable to any microorganism if single-crossover and Cre recombinase expression can be successfully used in the strain of interest.

## 5. Conclusions

In this study, an effective plasmid-curing method and an efficient large-size genome-deletion method was developed. The application of both methods will considerably advance genome manipulation in *Rhodococcus*, improving the stability of the genome and plasmids, and it will enable the elimination of unnecessary background host functions that retard the desirable activities of heterologous genes. These techniques may be applicable for strain improvement as well as for minimum genome studies on *Rhodococcus*, which have not been conducted previously.

## Figures and Tables

**Table 1:** Deletion size and genome size of wild and mutant strains of *R. erythropolis* JCM 2895.

Strain	Deletion Size (bp)	Total Deletion Size (bp)	Genome Size (bp)	Description
*R. erythropolis* JCM 2895	0	0	6,773,716	Wild-type, 1 chromosome and 4 plasmids
R0901	79,600	79,600	6,694,116	pR09C01 cured strain of JCM 2895
R0902	227,989	307,589	6,466,127	pR09L01 cured strain of R0901
R0903	5420	313,009	6,460,707	pREC01 cured strain of R0902
R0904	5444	318,453	6,455,263	pREC02 cured strain of R0903
R0905	117,555	436,008	6,337,754	T1 reduction strain of R0904
R0906	6428	442,436	6,331,332	T2 reduction strain of R0905
R0907	20,023	462,459	6,311,355	T3 reduction strain of R0906
R0908	45,236	507,695	6,266,177	T4 reduction strain of R0907
R0909	21,194	528,889	6,245,042	T5 reduction strain of R0908
R0910	45,770	574,659	6,199,331	T6 reduction strain of R0909
R0911	27,820	602,479	6,171,581	T7 reduction strain of R0910

## Data Availability

Not applicable.

## Supplementary Materials

The following supporting information can be downloaded at: https://www.mdpi.com/article/10.3390/microorganisms11020268/s1, Table S1: Bacterial strains and plasmids used in this study; Table S2: Primers used in this study; Figure S1: Determination of genome reduction target position (T1–T7) of *R. erythropolis* JCM 2895 by comparing genome sequence of four other *R. erythropolis* strains; Figure S2: Cre recombination between chromosomal *loxLE* and plasmid *loxRE*.
