# Supplementary material for: Development of Efficient Genome-Reduction Tool Based on Cre/loxP System in Rhodococcus erythropolis
Source: Microorganisms. 2023 Jan 19;11(2):268. doi: 10.3390/microorganisms11020268 (PMC9959502; doi:10.3390/microorganisms11020268)

Supplementary Materials

Development of efficient genome reduction tool based on Cre/*loxP* system in *Rhodococcus erythropolis*

by Watrau Kitagawa and Miyako Hata

Table S1. Bacterial strains and plasmids used in this study
Table S2. Primers used in this study
Figure S1. Determination of genome reduction target position (T1–T7) of *R. erythropolis* JCM 2895 by comparing genome sequence of four other *R. erythropolis* strains.
Figure S2. Cre recombination between chromosomal *loxLE* and plasmid *loxRE*.

Table S1. Bacterial strains and plasmids used in this study

Strain or plasmid	Relevant characteristic(s)	Reference or origin
Strains		
Rhodococcus strains		
R. erythropolis JCM 6824	Wild type, aurachin RE producer	Ref. 65 & 38
R. erythropolis DRE	Genome reduction derivative of JCM 6824	This study
R. erythropolis JCM 2895	Wild type, antibiotic protein producer, containing 4 plasmids; pR09L01, pR09C01, pREC01, and pREC02	Ref. 39 & 28
R. erythropolis R0901	pR09C01 cured strain of JCM2895	This study
R. erythropolis R0902	pR09L01 cured strain of R0901	This study
R. erythropolis R0903	pREC01 cured strain of R0902	This study
R. erythropolis R0904	pREC02 cured strain of R0903	This study
R. erythropolis R0905	Genome reduction derivative of R0904	This study
R. erythropolis R0906	Genome reduction derivative of R0905	This study
R. erythropolis R0907	Genome reduction derivative of R0906	This study
R. erythropolis R0908	Genome reduction derivative of R0907	This study
R. erythropolis R0909	Genome reduction derivative of R0908	This study
R. erythropolis R0910	Genome reduction derivative of R0909	This study
R. erythropolis R0911	Genome reduction derivative of R0910	This study
E. coli DH5α	Host strain for cloning of DNA	Nippon Gene
Plasmids		
pBluescriptII KS	Cloning vector, Amp^r	Stratagene
pBS-aphII-loxLE	pBluescriptII KS derivative, *loxLE*, Km^r	This study
pK18mobsacB	Cloning vector, *mob*, *sacB*, Km^r	Ref. 56
pK18mobsacB-loxLE	pK18mobsacB with *loxLE*	This study
pACYC184	Cloning vector, Cm^r, Tet^r	Ref. 58
pACYC-aac-loxRE	pACYC184 derivative, *loxRE*, Apr^r	This study
pCH-cre-loxRE	pACYC184 derivative, CH2.2 promoter (thiostrepton inducible), *cre*, *tipA*, *loxRE*, Apr^r, $Thio^r$	This study
pTip-QC2*	Expression vector for *Rhodococcus* sp., *tip* promoter (thiostrepton inducible), *tipA*, Cm^r, Ap^r, $Thio^r$	Ref. 41 & 44
pTip-sacB-cre*	pTip-QC2 derivative, *cre*, *sacB* (3rd vector)	This study
pTip-CH2.2*	Expression vector for *Rhodococcus* sp., CH2.2 promoter (thiostrepton inducible), *tipA*, Cm^r, Ap^r, $Thio^r$	Ref. 41
pTH18cs1::cre	Cloning vector, Cm^r, *cre*, used for *cre* source	Gift from Dr. H. Ikeda and Dr. M. Komatsu
pHN1237	Expression vector, Apr^r, used for Apr source	Ref. 59
pK18R09CPD1	pK18mobsacB with a DNA fragment of pR09C01	This study
pK18R09LPD01	pK18mobsacB with a DNA fragment of pR09L01	This study
pK18R09REC01D	pK18mobsacB with a DNA fragment of pREC01	This study
pK18R09REC02D	pK18mobsacB with a DNA fragment of pREC02	This study
pBS-D95k-LE	pBS-aphII-loxLE with a DNA fragment of JCM 6824 (left homolog)	This study
pAC-D95k-RE	pACYC-aac-loxRE with a DNA fragment of JCM 6824 (right homolog)	This study
pSLE-R09T1	pK18mobsacB-loxLE with left homolog of T1	This study
pCRE-R09T1	pCH-cre-loxRE with right homolog of T1	This study

Table S1. Bacterial strains and plasmids used in this study (continued)

Strain or plasmid	Relevant characteristic(s)	Reference or origin
pK18-R09T2	pK18mobsacB with left and right homolog of T2	This study
pSLE-R09T3	pK18mobsacB-loxLE with left homolog of T3	This study
pCRE-R09T3	pCH-cre-loxRE with right homolog of T3	This study
pSLE-R09T4	pK18mobsacB-loxLE with left homolog of T4	This study
pCRE-R09T4	pCH-cre-loxRE with right homolog of T4	This study
pSLE-R09T5	pK18mobsacB-loxLE with left homolog of T5	This study
pCRE-R09T5	pCH-cre-loxRE with right homolog of T5	This study
pSLE-R09T6	pK18mobsacB-loxLE with left homolog of T6	This study
pCRE-R09T6	pCH-cre-loxRE with right homolog of T6	This study
pSLE-R09T7	pK18mobsacB-loxLE with left homolog of T7	This study
pCRE-R09T7	pCH-cre-loxRE with right homolog of T7	This study

*Replicable vector in *Rhodococcus*

JCM, Japan Collection of Microorganisms

Amp^r, ampicillin resistance; Cm^r, chloramphenicol resistance; Tet^r, tetracycline resistance; Km^r, kanamycin resistance; Apr^r, Apramycin resistance; $Thio^r$, thiostrepton resistance.

References for Table S1. (Each reference number here is identical to that of main text)

65. Kitagawa, W.; Ozaki, T.; Nishioka, T.; Yasutake, Y.; Hata, M.; Nishiyama, M.; Kuzuyama, T.; Tamura, T. Cloning and heterologous expression of the aurachin RE biosynthesis gene cluster afford a new cytochrome P450 for quinoline N-hydroxylation. ChemBioChem 2013, 14, 1085-1093, doi:10.1002/cbic.201300167.

38. Kitagawa, W.; Hata, M.; Sekizuka, T.; Kuroda, M.; Ishikawa, J. Draft genome sequence of *Rhodococcus erythropolis* JCM 6824, an aurachin RE antibiotic producer. Genome Announcements 2014, 2, doi:10.1128/genomeA.01026-14.

39. Kitagawa, W.; Mitsuhashi, S.; Hata, M.; Tamura, T. Identification of a novel bacteriocin-like protein and structural gene from *Rhodococcus erythropolis* JCM 2895, using suppression-subtractive hybridization. J. Antibiot. 2018, 71, 872-879, doi:10.1038/s41429-018-0078-3.

28. Kitagawa, W.; Hata, M. Complete genome sequence of *Rhodococcus erythropolis* JCM 2895, an antibiotic protein-producing strain. Microbiol Resour Announc 2022, e0068222, doi:10.1128/mra.00682-22.

56. Schafer, A.; Tauch, A.; Jager, W.; Kalinowski, J.; Thierbach, G.; Puhler, A. Small mobilizable multi-purpose cloning vectors derived from the *Escherichia coli* plasmids pK18 and pK19: selection of defined deletions in the chromosome of *Corynebacterium glutamicum*. *Gene* 1994, *145*, 69-73.

58. Chang, A.C.; Cohen, S.N. Construction and characterization of amplifiable multicopy DNA cloning vehicles derived from the P15A cryptic miniplasmid. *J. Bacteriol.* **1978**, *134*, 1141-1156, doi:10.1128/jb.134.3.1141-1156.1978.

41. Nakashima, N.; Tamura, T. A novel system for expressing recombinant proteins over a wide temperature range from 4 to 35 degrees C. Biotechnol. Bioeng. 2004, 86, 136-148.

44. Nakashima, N.; Tamura, T. Isolation and characterization of a rolling-circle-type plasmid form *Rhodococcus erythropolis* and application of the plasmid to multiple-recombinant-protein expression. Appl. Environ. Microbiol. 2004, 70, 5557-5568.

59. Nakashima, N.; Tamura, T. Conditional gene silencing of multiple genes with antisense RNAs and generation of a mutator strain of *Escherichia coli*. Nucleic Acids Res. 2009, 37, doi:ARTN e103 10.1093/nar/gkp498.

Table S2. Primers used in this study

Primer	Usage	Sequence (5′–3′)
R09CPD-F1	Construction of pK18R09CPD1	ATATAGAATTCCGTTGAAGGTGGAAGCGAC
R09CPD-R1	Construction of pK18R09CPD1	TATTATCTAGACGGGTATTGCAGCGATTCC
R09LPD-F01	Construction of pK18R09LPD01	ATATAGAATTCTTCTGGGAGTTCGTCGACC
R09LPD-R01	Construction of pK18R09LPD01	TATTATCTAGACATCAAGCGCTCCACATCC
REC01D-F	Construction of pK18R09REC01D	ATATAGAATTCGAGGCCTGACCTAGAAACTCC
REC01D-R	Construction of pK18R09REC01D	TATTATCTAGAAACAAAGCCATATCAGGTATCC
REC02D-F	Construction of pK18R09REC02D	ATATAGAATTCATGACTGGACCACAGGAGAG
REC02D-R	Construction of pK18R09REC02D	TATTATCTAGACTGTGACTCAGTTCACACCTGC
95k-LE-F2	Construction of pBS-D95k-LE	TCAAGTTCTAGAGCAAGAAGATCAAGCCCGAG
95k-LE-R	Construction of pBS-D95k-LE	TCAAGTGCATGCCGCTCTGTTGACCATGATG
95k-RE-F	Construction of pAC-D95k-RE	TCAAGTACTAGTGATACCGAGTGCAGCAAG
95k-RE-R2	Construction of pAC-D95k-RE	TCAAGTCCTGCAGGAACATGCAAAGCCACCGTAG
R09T1-1F	Construction of pSLE-R09T1	AAAAAATCTAGAGGGCAATCAAGTCGACGATG
R09T1-1R	Construction of pSLE-R09T1	AAAAAAAAGCTTCATGTGCACTACCAGATCGC
R09T1-2F	Construction of pCRE-R09T1	AAAAAAACTAGTACCCATTCTGTCCGGTATCC
R09T1-2R	Construction of pCRE-R09T1	AAAAAACCTGCAGGTCGGAAGGAGTGAACTGGTC
R09T2-1F	Construction of pK18-R09T2 and double crossover detection	AAAAAAGAATTCGCGACGATCCACTGATTCAC
R09T2-1R	Construction of pK18-R09T2	AAAAAATCTAGACAGCGCAGAATAAGTGGTCC
R09T2-2F	Construction of pK18-R09T2	AAAAAATCTAGAGACACAATAGGCTGCTCACG
R09T2-2R	Construction of pK18-R09T2 and double crossover detection	AAAAAAAAGCTTCGGATGGATCAGATCAACGC
R09T3-1F	Construction of pSLE-R09T3	AAAAAATCTAGACACTGAAATGGGTGTGGCTC
R09T3-1R	Construction of pSLE-R09T3	AAAAAAAAGCTTCACGACCAGAGCAATCGATC
R09T3-2F	Construction of pCRE-R09T3	AAAAAAACTAGTATGGAAGCGTGTCATCGTTG
R09T3-2R	Construction of pCRE-R09T3	AAAAAACCTGCAGGCAACTGATTCCGACGGCATC
R09T4-1F	Construction of pSLE-R09T4	AAAAAATCTAGAAAGAGAATCATCAACGCGCC
R09T4-1R	Construction of pSLE-R09T4	AAAAAACTGCAGCCAGCGGTGATATCCAAACG
R09T4-2F	Construction of pCRE-R09T4	AAAAAAACTAGTAAGCAGCGTTGAGAATTCCC
R09T4-2R	Construction of pCRE-R09T4	AAAAAACCTGCAGGCCGAAATTCCCGACGTCATG
R09T5-1F	Construction of pSLE-R09T5	AAAAAATCTAGATCGATTGTCGTCGAACCCTG
R09T5-1R	Construction of pSLE-R09T5	AAAAAACCTGCAGGACCTCACTCAAATCCCGCAC
R09T5-2F	Construction of pCRE-R09T5	AAAAAAACTAGTTCAAGCCCGTCCCTGAAATC
R09T5-2R	Construction of pCRE-R09T5	AAAAAACCTGCAGGAAGAGTGGCACGAACCTGA
R09T6-1F	Construction of pSLE-R09T6	AAAAAATCTAGACGACCGTTTCATCGCGAATC
R09T6-1R	Construction of pSLE-R09T6	AAAAAACCTGCAGGGATGTACTCGAGCTCACCCG
R09T6-2F	Construction of pCRE-R09T6	AAAAAAACTAGTTGTTGGTGCTTTGTTGCTCG
R09T6-2R	Construction of pCRE-R09T6	AAAAAACCTGCAGGAGTGCACTCGCTCATCAGTC
R09T7-1F	Construction of pSLE-R09T7	AAAAAAGAATTCGAGAAGAGGATCGCGGTGAG
R09T7-1R	Construction of pSLE-R09T7	AAAAAATCTAGACCCGATAGCTCCAGAACGAC
R09T7-2F	Construction of pCRE-R09T7	AAAAAAACTAGTCTTGGCGCCGTACATCATTG
R09T7-2R	Construction of pCRE-R09T7	AAAAAAACTAGTGTCCCAAGGCTCCATGAGTG
aphII-UR	Single crossover detection, hybridize to kanamycin resistant gene (*aphII*)	ATCCATCTTGTTCAATCATGCG
R09CPD-SCC1	pK18R09CPD1 single crossover detection	GGATGCAGTCAAGATCGTCG
R09CPD-F2	pR09C01 detection	ATATAGAATTCTTCCACTTGACGTCCTCCC

Table S2. Primers used in this study (continued)

Primer	Usage	Sequence (5′–3′)
R09CPD-R2	pR09C01 detection	TAATATCTAGACCACACCAGCATCAACACC
R09LPD-SCCF1	pK18R09LPD1 single crossover detection	GGAAACGGGAGGTGTTGTG
R09LPD-F02	pR09L01 detection	ATATAGAATTCAACTTCGGGACTCCATGGG
R09LPD-R02	pR09L01 detection	TATTATCTAGACGAACCATTCCACGCTCAC
R09REC01-SCCF	pK18R09REC01D single crossover detection	GCCTGTTCTTCCTCAGCAAC
REC01-F02	pREC01 detection	GCGTCGGACTCGAATAGTTG
REC01-R02	pREC01 detection	TCTCGCATCGTATGTTCGTC
R09REC02-SCCF	pK18R09REC02D single crossover detection	TTATGCGATGCGACTTTGTC
REC02-F02	pREC02 detection	GCAGTTCCAGAAGCAGAAGG
REC02-R02	pREC02 detection	TTTACGGATCGCTTGTTTCC
D95k-LE-CF	pBS-D95k-LE single crossover detection	ACACCTACGACATCAGCGAC
LE-DR	pBS-D95k-LE, pSLE-R09T1, pCRE-R09T3, pSLE-R09T4, pSLE-R09T5, pSLE-R09T6, pSLE-R09T7 single crossover detection	CTGCAAGGCGATTAAGTTGGG
RE-UF	pAC-D95k-RE single crossover detection	CCAGCAATAGACATAAGCGGC
D95k-RE-CR	pAC-D95k-RE single crossover detection	GAGGGTCTTCGTGTTGTGTG
R09T1-FF	pSLE-R09T1 single crossover detection and T1 reduction detection	CGACAAGGCGAAGTGGTATG
pTCLR-2917-F	pCRE-R09T1, pCRE-R09T3, pCRE-R09T4, pCRE-R09T5, pCRE-R09T6, pCRE-R09T7 single crossover detection	TGGGCGAGATGTACGTGTC
R09T1-RR	pCRE-R09T1 single crossover detection and T1 reduction detection	GTCATCCGAATCTTGCTCCG
18MCS-FF	pk18-R09T2 single crossover detection	CTTCCGGCTCGTATGTTGTG
R09T2-singleC1	pk18-R09T2 single crossover detection	AAACATCGCAGCTTCCCTTG
R09T2-singleC2	pk18-R09T2 single crossover detection	TTGATTGCCCAACTGTCAGC
R09T3-FF	pSLE-R09T3 single crossover detection and T3 reduction detection	TGATGATTCTCGCCGCCTAC
R09T3-RR	pCRE-R09T3 single crossover detection and T3 reduction detection	GATTCTGGGAACGCGACTTC
R09T4-FF	pSLE-R09T4 single crossover detection and T4 reduction detection	CAACGATGAACACACCCTGG
R09T4-RR	pCRE-R09T4 single crossover detection and T4 reduction detection	GATTTCGAACCAAGCCCGG
R09T5-FF	pSLE-R09T5 single crossover detection and T5 reduction detection	TCGAAGTTGAAGTCTCCGCC
R09T5-RR	pCRE-R09T5 single crossover detection and T5 reduction detection	AGTTCACGTACTACCGCGTC
R09T6-FF	pSLE-R09T6 single crossover detection and T6 reduction detection	GGTTACAGCCAGTCCCAGTC
R09T6-RR	pCRE-R09T6 single crossover detection and T6 reduction detection	TGGACATCGTCGGTCATTCC
R09T7-FF	pSLE-R09T7 single crossover detection and T7 reduction detection	TGATCTGCGTCAGGTGATCG
R09T7-RR	pCRE-R09T7 single crossover detection and T7 reduction detection	TCTTCGGTGATCCACAGACG

Figure S1. Determination of genome reduction target position (T1–T7) of *R. erythropolis* JCM 2895 by comparing genome sequence of four other *R. erythropolis* strains. Chromosomal DNA sequence of JCM 2895 (horizontal axis) was compared with JCM 6825, NBRC 100887 (PR4), DSM 11397, and JCM 6824 (vertical axis) by GenomeMatcher software. The figures of independent comparison were overlayed to identify JCM 2895 specific DNA region among them. The red line indicates identical/nearly identical region between them, and horizontal break indicates JCM 2895 specific DNA region. The specific DNA region over 5-kb in size were selected for the target of genome reduction (T1–T7).

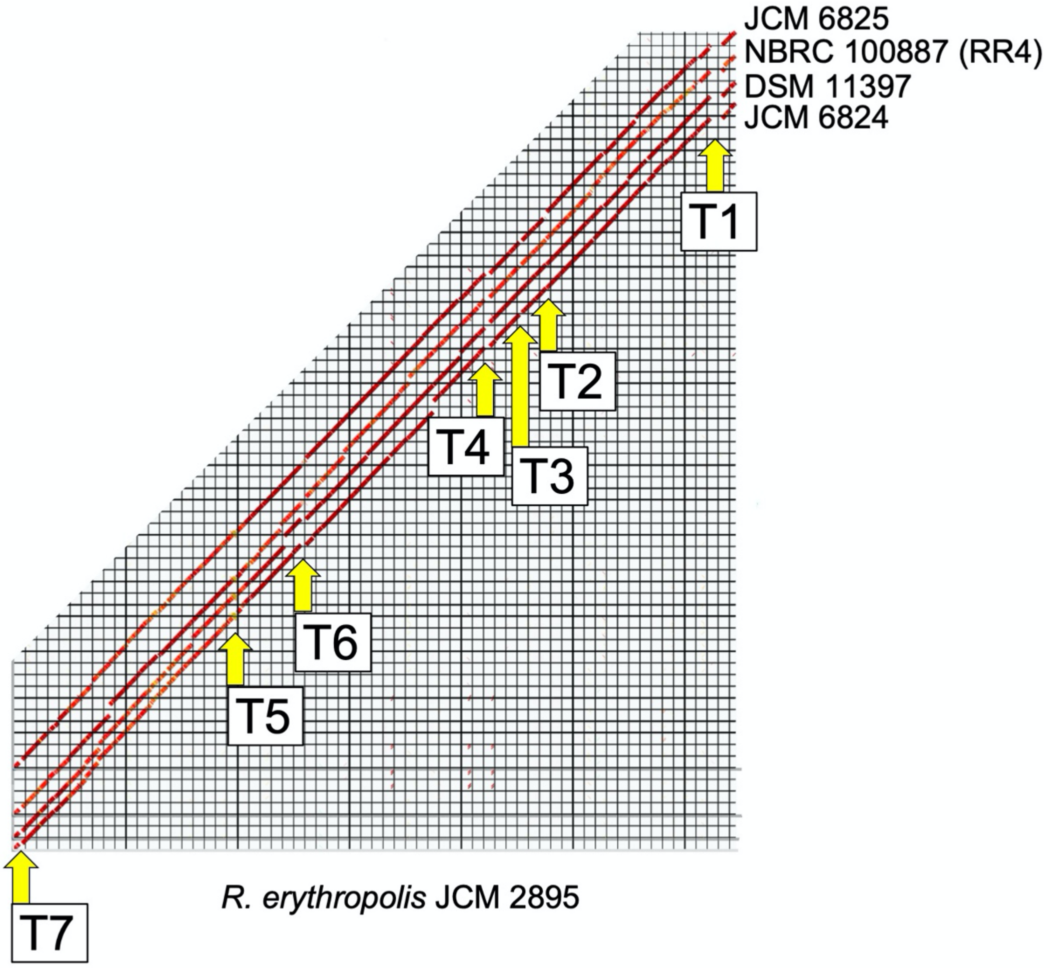

Figure S2. Cre recombination between chromosomal *loxLE* and plasmid *loxRE*. If a leaky promoter was used for *cre* expression, even if the level was very low, the recombination would happen between chromosome and plasmid, before the single crossover of 2nd vector, since the Cre recombinase is highly active. In that case, the whole 2nd vector DNA sequence would be integrated at *loxLE* site, and the genome reduction would end in failure. The two lox mutant sequence, *loxLR* and *loxRL* (the hybrid sequence of *loxLE* and *loxRE*) remains on the chromosome.

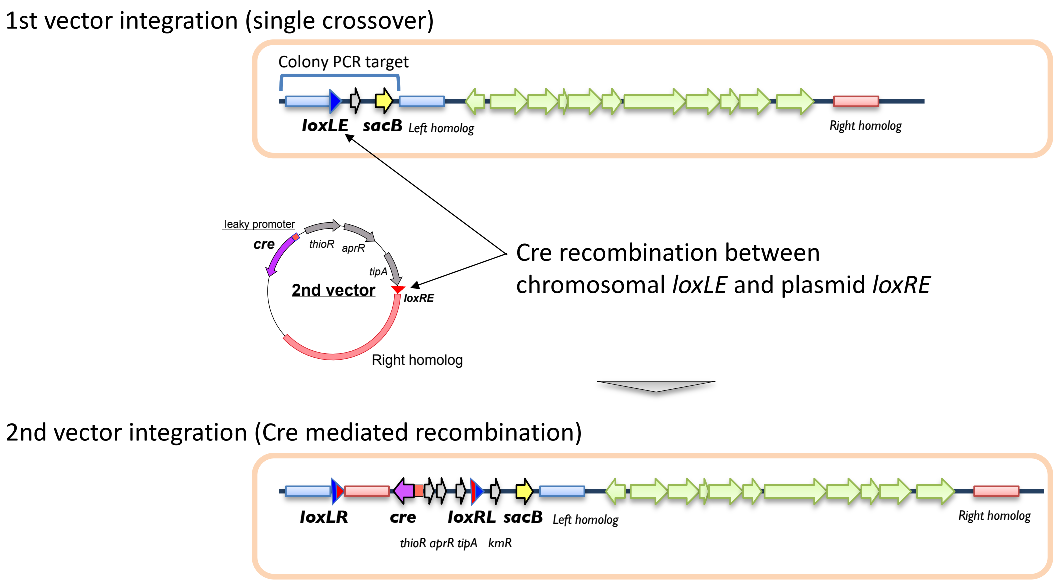

Supplement: Supplementary file 1 [file microorganisms-11-00268-s001.zip › microorganisms-2161690-supplementary.pdf]
